# A Lifetime of Double Vision: A Case Report and Literature Review of Chronic Strabismus

**DOI:** 10.7759/cureus.9607

**Published:** 2020-08-07

**Authors:** Jonathan H Desimone, Tiffany Wood, Devon C De Simone, Kelly DeSimone

**Affiliations:** 1 Department of Research, The Alabama College of Osteopathic Medicine, Dothan, USA; 2 Department of Vision Therapy, Eye Priority Re-Vision, Phoenix, USA

**Keywords:** strabismus, diplopia, double vision, vision therapy, neuro-optometric rehabilitation, strabismus surgery, eye turn, crossed eye, lazy eye, modern strabismus treatment

## Abstract

Strabismus is a condition preventing foveal alignment in normal gaze. Its effects can have long-standing impacts on physical, psychological, and social health. Multiple treatment options exist for strabismus such as surgery, optical correction, and vision therapy, however, treatment efficacy remains variable with little consensus on long-term impacts. Diplopia is a common side effect of the ocular misalignment secondary to strabismus and its treatment. When defining long-term success of treating strabismus, it is essential that visual sensory perception and binocular fusion has been integrated and remedied. Strabismus is complex and can be challenging to manage. To illustrate this, we report a 67-year-old female who presented with complications from long-term treatment of infantile strabismus. This case highlights differences in historical and contemporary strabismus treatments.

## Introduction

Strabismus, commonly referred to as an eye turn, crossed eye, lazy eye, wandering eye, squint, etc. is a common childhood problem that can be hereditary, occurring in 2.1% to 3.6% of the population [1-3]. This vision condition characterizes an anomaly in which the eyes are unable to align. Classification of strabismus is based on the direction of deviation, frequency of deviation, and the amount of deviation between eyes. Direction of deviation is categorized as either inward (eso), outward (exo), upward (hyper), downward (hypo) or torsional [4]. Frequency of deviation is categorized as either a tropia or a phoria. A tropia occurs when the deviation is visible under binocular viewing conditions (manifest strabismus). A phoria occurs when the deviation is not visible with binocular viewing, but presents with disruption to the binocular system (latent strabismus) [4]. The difference between eye deviation is either comitant (concomitant) where the deviation between eyes remains the same over time and change in gaze or incomitant (nonconcomitancy) where the deviation between the eyes change either over time or with a change in gaze [4]. Diagnosis and classification of strabismus are determined via the cover test [5]. In order to quantify the deviation in the affected eye, prisms are used to measure the angle of deviation in diopters (D) [6].

While multiple treatment options exist to manage strabismus, the efficacy remains variable with a lack of consensus regarding long-term impacts. Nevertheless, it is important to consider treatment options on an individual basis because of the complex nature of strabismus and its associated visual impairments [4]. Despite the cause and severity, the ideal goal for full remediation is to obtain functional binocularity and persisting favorable cosmesis [4]. On a case-by-case basis, these goals may vary with respect to patient needs and wants. When considering the long-term success post-treatment of strabismus, it is essential that visual perception and processing have been remedied through the integration of sensory and motor fusion. Here, we illustrate the historical standard of care for strabismus and provide our recommended treatment plan for the patient with strabismus. This case report documents the experiences of a patient who suffered from infantile esotropia, receiving a wide variety of treatments over the course of her life.

## Case presentation

A 67-year-old female presented to the optometry clinic 14 years ago requesting new glasses for double vision. The patient's left eye was dominant. She had learned to suppress vision in the right eye to avoid diplopia. Brief episodes of diplopia were tolerated. Diplopia was made better by closing her right eye, tilting her head, consciously suppressing her right eye, and body posture accommodations to improve her vertical alignment. The diplopia was worse with reading, looking to the left with eyes only, and with minor glasses adjustments. Associated symptoms included blurred vision at a distance, watery eyes, dry eyes, bilateral eye pain, longstanding headaches, lack of depth perception, impaired night vision, poor general coordination, and a migraine cycle instigated by the onset of diplopia that varies from tolerable to complete debilitation.

An extensive investigation into her past medical history revealed a lifetime of attempted treatments for esotropia. At two years old, she received her first strabismus surgery. Due to surgery regression, two additional strabismus surgeries were performed at the ages of 10 and 16. At the age of 23, double vision presented and was treated with vision therapy. Continuous vision therapy and optical correction (use of lenses with added powers, prism, binasal occlusion, contacts, etc.) were attempted unsuccessfully for many years. After approximately 20 years, suppression of binocular vision via optical blur was used and reportedly provided palliative care. Considering her family history, her only son also had strabismus from birth that was successfully treated with a combination of early vision therapy and strabismus surgery.

On examination, the patient was a pleasant animated female who moved her head excessively while talking. Cosmetically, no eye turn is apparent; however, the patient has a head tilt to the left with the face rotated to the right. Visual acuity with habitual glasses is OD +2.25, 20/30 and OS +2.50, 20/20-. Refraction results are OD + 4.25-1.00 x 130, 20/20- and OS +3.25-0.75 x 95, 20/20. Patient has a 12D constant right esotropia with 1D constant right hypertropia both at distance and near. An anterior eye segment exam revealed a mild nuclear sclerotic cataract in both eyes (OU), meibomian gland disruption, and reduced tear prism. A posterior eye segment exam was unremarkable.

The patient was diagnosed with refractive, binocular, and ocular health conditions. Refractive diagnoses were latent hyperopia OU, astigmatism OU, and presbyopia. The treatment plan involved optically aided suppression via progressive lenses with an anti-reflective coating (OD +2.25 OS +2.50 with a +2.00 add). The patient was unable to tolerate a single vision or any lens changes in the office as it interfered with her optically induced suppression. Binocular diagnoses were constant right esotropia, constant right vertical heterophoria, diplopia, and suppression of binocular vision (right eye suppression). At this time, the patient was not interested in additional remediation beyond the optically aided suppression and refused the use of prism due to past experiences. The ocular health diagnoses were dry eyes (Keratitis Sicca), seasonal allergic conjunctivitis, and an age-related cataract. Over the counter eye drops were recommended as needed for dry eyes. The patient was monitored for changes in age-related cataracts during subsequent office visits.

Over time, the patient returned to the clinic with issues of dry eyes and her diplopia was exacerbated by typical refractive changes. At each dispense of new glasses, the patient required excessive time for frame adjustments. This progressed over the next 14 years, at which point her double vision and headaches began to increase. The patient’s cataracts matured, she required a hired driver, and had impaired mobility after a recent fall. Cataracts made diplopia management even more difficult because the patient’s right eye began to see better than her dominant left eye. She could no longer suppress her right eye. Co-management and patient support with an ophthalmologist resulted in a successful left eye cataract surgery. After the first successful procedure on the left eye, the patient was hesitant to have surgery on her right eye as suppression was once again possible. Brief diplopia persists in left gaze and she will continue to need long term care with corrective lenses.

## Discussion

This patient’s case is one of infantile strabismus without amblyopia, initially treated with three strabismus surgeries. While the surgeries made the eyes cosmetically straight, they did not address visual sensory perception and binocular fusion which contributed to the patient developing diplopia. Had the doctors been collaborating with vision therapy, this outcome may not have manifested. After the development of diplopia, the patient then pursued vision therapy on her own accord. Due to incorrect historical beliefs of critical periods in visual development [7,8], the patient’s therapy was discontinued because of her age before its benefit could be actualized. Corrective lenses, prism, and contact lenses did not resolve binocular disruption. Finally, and as a last resort, optical aided suppression provided palliative care and has since been the mainstay of her treatment.

**Figure 1:**
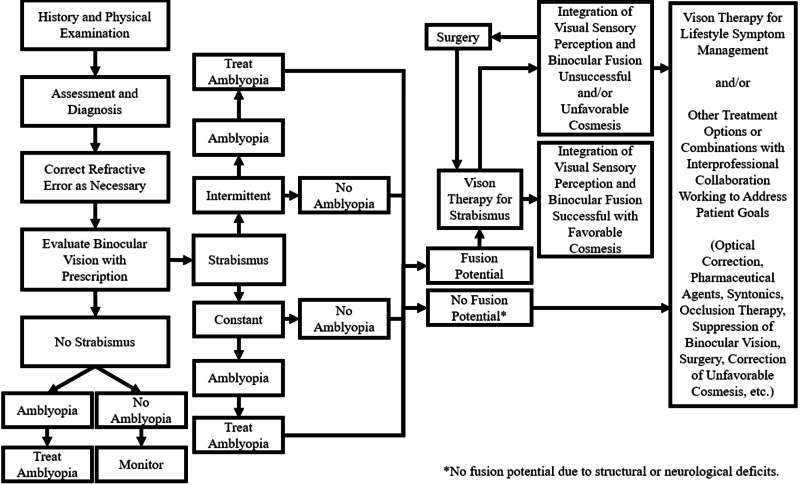
Recommended treatment plan of the patient with strabismus A treatment algorithm for treating patients with strabismus. Treatment begins with a thorough history and physical examination then progresses through a decision tree for optimum treatment.

Treatment for strabismus should begin with a baseline evaluation to identify the optimal treatment plan. Early intervention is ideal. Certainly, the patient received early treatment, but the sequence and lack of collaboration between her doctors is what contributed to her diplopia and further complicated her case. Our recommended treatment plan for all patients with strabismus to reduce complications is identified in the embedded figure (Figure 1). Treatment options include optical correction (such as the use of lenses with added powers, prism, and contacts), pharmaceutical agents, balancing the autonomic nervous system with optometric phototherapy (syntonics), binocular vision therapy, occlusion therapy, and surgery. When considering long-term success for the patient, it is essential that integration of visual sensory perception and binocular fusion occurs to restore the patient’s ability to perceive and process the visual scene in addition to favorable cosmesis.

Ideally. vision therapy would begin prior to any surgical intervention with flexible goals dependent upon patient needs. Surgery may not even be necessary as strabismus and its side effects can sometimes be remedied via vision therapy alone. Additionally, surgery may interfere with proprioceptive feedback from the ocular muscles which could make rehabilitation even more difficult if neuro-visual connections were not previously established via vision therapy [9].

Considering the age at which a patient can benefit from vision therapy. In the early 1960s, belief of critical periods in brain development influenced the treatment of strabismus as early as possible for success to be feasible [7]. However, newfound research supporting ongoing neuroplasticity disproves this belief with documented adult successes in vision therapy [8]. Success at any age is feasible.

How does vision therapy work for treating the patient with strabismus? Vision therapy integrates the neuro-visual pathways necessary for full visual function. Vision therapy does this by addressing levels of oculomotor control, awareness of periphery, balanced accommodative ability, elimination of suppression, perception of stereopsis, vergence flexibility, improved self-image, and improved visual quality of life [10,11]. The length of vison therapy treatment varies from months to years based on each unique case.

If the patient reaches a plateau in their vision therapy program, then a surgical consultation is warranted. After surgery, vision therapy should follow for full neuro-visual rehabilitation to reestablish proprioceptive feedback for ocular motor control and binocular vision as well as to ensure that visual sensory perception and binocular fusion integration has occurred. For comparison, consider a patient with knee problems. Most likely, they would initially be referred for physical therapy. If a plateau in therapy was reached, there is a good chance they would be referred for surgery. Following knee surgery, additional physical therapy for full rehabilitation to minimize the chance of complications would be expected.

## Conclusions

Strabismus presents unique challenges for treatment. This case highlights the differences in historical and contemporary strabismus treatments. For this patient, it is likely strabismus surgery contributed to the development of diplopia due to lack of collaboration with vision therapy, which complicated further management. Ideally, vision therapy would be initiated prior to surgery. If vision therapy reaches a plateau, then a surgical consult is warranted. Following surgery, vision therapy would be continued to ensure visual sensory perception and binocular fusion integration.
